# Bioinformatics in Italy: BITS2006, the third annual meeting of the Italian Society of Bioinformatics

**DOI:** 10.1186/1471-2105-8-S1-S1

**Published:** 2007-03-08

**Authors:** Rita Casadio, Manuela Helmer-Citterich, Graziano Pesole

**Affiliations:** 1 Department of Biology, University of Bologna, Italy; 2 Centre for Molecular Bioinformatics, Dept of Biology "Enrico Calef", University of Rome Tor Vergata, Rome, Italy; 3 Dept of Biochemistry and Molecular Biology "Ernesto Quagliariello", University of Bari, Italy

## Overview

The Italian Society of Bioinformatics (BITS, ) was founded in 2003, after five years of constructive and pleasant informal meetings. Since then, there has been a growing interest in the discipline that involves Italian groups working in different fields, including molecular biology, genetics, structural biochemistry, mathematics, physics and informatics. The 3^rd ^Annual BITS Meeting, , was held in Bologna, Italy, on April 28-29 2006, in a beautiful historical building, the Convento di San Domenico. Over 200 scientists actively working in the field or strongly interested in its development met and discussed their work, state of the art and future perspectives. A total of 115 abstracts were accepted and presented in the poster session and 24 of them were selected for oral presentation.

Distinguished scientists were invited to give lectures at the BITS 2006 Conference: Alfonso Valencia, Spanish National Cancer Research Center, Madrid, gave the "Giuliano Preparata" Lecture. Anna Tramontano, University of Rome "La Sapienza", Dominic Clark and Dietrich Rebholz-Schuhmann, both at EBI, Hinxton, were invited as keynote speakers.

A round table discussion on the most timely and important problems of Bioinformatics and its development in Italy was also held. Participants were: Cecilia Saccone (CNR/University of Bari and honorary President of BITS), Lilia Alberghina (University of Milano Bicocca), Alberto Albertini (CNR ITB, Milano), Aldo Tagliabue (ALTA/Chiron Siena), Corrado Priami (Centre for Computational and Systems Biology, University of Trento), Enrico Nardelli (Computer Science, University of Roma "Tor Vergata").

The conference was organized into six thematic sessions: i) Genome Screening and Prediction; ii) Computational Proteomics; iii) Microarray and Genome Analysis; iv) Protein Structure and Function; v) Genomes and Evolution; and vi) Networks and Systems Biology.

From these conference sessions, 36 papers were submitted for publication in this *BMC Bioinformatics *supplement. We adopted a stringent reviewing procedure, based on selected Associated Editors handling the process according to their recognized knowledge in specific subjects of Bioinformatics and following a peer review procedure.

After this process, 23 papers were accepted. They cover different aspects of theoretical and applied Bioinformatics, such as: sequence and protein analysis, analysis of gene expression, molecular evolution, databases, informatics tools for biology, text mining and systems biology.

Whether this will help in encouraging scientists involved in biological and/or medical researches in our country to plan, merge and spread their knowledge by means of Bioinformatics is left to the future and to the availability of national grants specifically devoted to this.

In this presentation, we gratefully acknowledge the patience and help of our colleagues around the world who helped in the process of reviewing as Associate Editors:

Alex Bateman, Wellcome Trust Sanger Institute, UK

Alvis Brazma, European Bioinformatics Institute, UK

Peter Clote, Boston College, USA

Joaquin Dopazo, Centro de Investigación "Principe Felipe", Valencia, Spain

Dan Gusfield, Department of Computer Science, UC, Berkley, USA

Des Higgins, University College Dublin, Ireland

Ivo Hofacker, Institute for Theoretical Chemistry, University Vienna, Austria

David Jones, University College, London, UK

Misha Kapushesky, European Bioinformatics Institute, UK

Reinhard Laubenbacher, Virginia Bioinformatics Institute at Virginia Tech, Virginia, USA

Stefano Lonardi, Department of Computer Science and Engineering, University of California at Riverside, USA

Wojciech Makalowski, Penn State University, PA, USA

Arthur Lesk, Penn State University, PA, USA

Giovanni Parmigiani, The Sidney Kimmel Comprehensive Cancer Center at Johns Hopkins University, Baltimore, MD, USA

Peter Rice, European Bioinformatics Institute, UK

Marie-France Sagot, BAOBAB Team, INRIA Université Claude Bernard, Lyon I, France

Luis Serrano, EMBL, Heidelberg, Germany

Alfonso Valencia, Centro Nacional de Investigaciones Oncológicas, Madrid, Spain

## Next meeting

The next Annual meeting of the Italian Society of Bioinformatics will be held in Naples, April 26–28, 2007. Further information about BITS2007 will be available on our web site at the address .

